# Understanding the financial cost of cancer clinical trial participation

**DOI:** 10.1002/cam4.7185

**Published:** 2024-04-17

**Authors:** Courtney P. Williams, Luqin Deng, Nicole E. Caston, Kathleen Gallagher, Rebekah Angove, Maria Pisu, Andres Azuero, Rebecca Arend, Gabrielle B. Rocque

**Affiliations:** ^1^ University of Alabama at Birmingham Birmingham Alabama USA; ^2^ Patient Advocate Foundation Hampton Virginia USA

**Keywords:** cancer, clinical trials, costs, equity, financial hardship

## Abstract

**Background:**

Though financial hardship is a well‐documented adverse effect of standard‐of‐care cancer treatment, little is known about out‐of‐pocket costs and their impact on patients participating in cancer clinical trials. This study explored the financial effects of cancer clinical trial participation.

**Methods:**

This cross‐sectional analysis used survey data collected in December 2022 and May 2023 from individuals with cancer previously served by Patient Advocate Foundation, a nonprofit organization providing social needs navigation and financial assistance to US adults with a chronic illness. Surveys included questions on cancer clinical trial participation, trial‐related financial hardship, and sociodemographic data. Descriptive and bivariate analyses were conducted using Cramer's *V* to estimate the in‐sample magnitude of association. Associations between trial‐related financial hardship and sociodemographics were estimated using adjusted relative risks (aRR) and corresponding 95% confidence intervals (CI) from modified Poisson regression models with robust standard errors.

**Results:**

Of 650 survey respondents, 18% (*N* = 118) reported ever participating in a cancer clinical trial. Of those, 47% (*n* = 55) reported financial hardship as a result of their trial participation. Respondents reporting trial‐related financial hardship were more often unemployed or disabled (58% vs. 43%; *V* = 0.15), Medicare enrolled (53% vs. 40%; *V* = 0.15), and traveled >1 h to their cancer provider (45% vs. 17%; *V* = 0.33) compared to respondents reporting no hardship. Respondents who experienced trial‐related financial hardship most often reported expenses from travel (reported by 71% of respondents), medical bills (58%), dining out (40%), or housing needs (40%). Modeling results indicated that respondents traveling >1 h vs. ≤30 min to their cancer provider had a 2.2× higher risk of financial hardship, even after adjusting for respondent race, income, employment, and insurance status (aRR = 2.2, 95% CI 1.3–3.8). Most respondents (53%) reported needing $200–$1000 per month to compensate for trial‐related expenses. Over half (51%) of respondents reported less willingness to participate in future clinical trials due to incurred financial hardship. Notably, of patients who did not participate in a cancer clinical trial (*n* = 532), 13% declined participation due to cost.

**Conclusion:**

Cancer clinical trial‐related financial hardship, most often stemming from travel expenses, affected almost half of trial‐enrolled patients. Interventions are needed to reduce adverse financial participation effects and potentially improve cancer clinical trial participation.

## 1 BACKGROUND

Cancer clinical trials test the safety and efficacy of new therapies, technologies, interventions or new delivery, dosing, or sequencing of approved therapies to prevent, detect, or treat cancer. Patients who participate in clinical trials receive high‐quality cancer care, since they receive access to novel, cutting‐edge treatments before they are more widely available, increased treatment options if unresponsive to standard‐of‐care treatments, highly monitored care by a clinical research team, and aid in the advancement of cancer treatment and care to help patients like themselves in the future.^1^, ^2^, ^3^, ^4^, ^5^ However, due to structural, clinical, and attitudinal barriers, several patient population subgroups are underrepresented in trials, including patients who are minoritized, older, experiencing multiple comorbid conditions, or residing in geographically rural locations.^6^, ^7^, ^8^, ^9^ Previous studies have found individuals with lower incomes are another patient population subgroup less likely to participate in clinical trials.^10^, ^11^ Individual financial status may play a key role in driving cancer clinical trial participation inequities, since participation in cancer clinical trials is often coupled with high out‐of‐pocket costs. Though the investigational agent is commonly covered by the trial sponsor, direct research costs that are not considered standard of care are uncovered by both the trial sponsor and patients' insurance, leaving the patient responsible for a large portion, if not all, of their care costs.^12^ This could include the standard‐of‐care component of the treatment regimen, trial‐related labs, imaging, procedures, or out‐of‐network care, as well as indirect costs of participation, such as travel or time off work.^13^, ^14^, ^15^, ^16^ Because of these uncovered costs, it is estimated that almost half of patients in early phase oncologic clinical trials spend at least $1000 per month out‐of‐pocket to participate.^17^

Our previous study of >3500 US adults nationwide found that respondents with lower perceived income had double the odds of reporting a cost‐related consideration as very influential to clinical trial participation than those with higher perceived income, even after accounting for patient age, race, and residence.^18^ However, respondents from this study were disease‐agnostic and were considering hypothetical clinical trial participation. Little is known about trial‐related financial hardship, defined as high out‐of‐pocket costs and accompanying financial distress,^19^ and its impact on patients with financial barriers to care considering or participating in cancer‐specific clinical trials. Therefore, this study explored the financial effects of cancer clinical trial participation in individuals with cancer who have experienced cost‐related barriers to cancer care.

## 2 METHODS

### 2.1 Study design and sample

This cross‐sectional analysis used survey data collected in December 2022 and May 2023 from individuals with cancer previously served by Patient Advocate Foundation (PAF). PAF is a nonprofit organization providing social needs navigation and financial assistance to individuals with a complex or chronic illness within the United States.^20^ Surveys were distributed via email to adults with a previous cancer diagnosis who received PAF services from January 2020 to December 2021. The survey consisted of 25 questions eliciting individual experiences with cancer clinical trial participation, cancer clinical trial‐related financial hardship, cancer characteristics, and sociodemographics (Figure S1). Individuals were invited to participate via e‐mail and received up to three reminder emails for participation. Inclusion criteria included a valid email address, aged 18 or older, English reading proficiency, US residence, and a previous cancer diagnosis. Informed consent was obtained electronically. Five $100 gift cards were offered as a random drawing for participation. This study was considered exempt by the University of Alabama at Birmingham Institutional Review Board (IRB‐300010015).

### 2.2 Outcomes: Cancer clinical trial experiences

Survey respondents were asked if they had ever participated in any cancer‐specific clinical trial. If so, respondents were then asked a series of questions about trial‐related financial hardship. The primary study outcome was a subjective measure of financial hardship, wherein respondents were asked if participating in a cancer clinical trial resulted in financial hardship for them and their family (not at all vs. a little bit, somewhat, quite a bit, very much). For clarity, examples of high out‐of‐pocket costs or worry about finances were included with the survey question (Figure S1). To understand objective measures of trial‐related financial hardship, respondents were also asked about the types of trial‐related expenses that were a financial hardship (travel, food, child/adult care, employment, medical bills/insurance, and lodging/basic needs), and the monthly amount of financial assistance that would have compensated for trial‐related medical and nonmedical expenses (<$200, $200–500, $501–1000, $1001–2000, $2001–4000, and >$4000). Respondents were also asked to report how trial‐related financial hardship affected their willingness to participate in future clinical trials (much less likely, less likely, and would not affect decision). If the respondent had never participated in a cancer clinical trial, they were asked if they declined enrollment due to the costs of participating (yes, no).

### 2.3 Respondent sociodemographic and clinical characteristics

Self‐reported sociodemographic characteristics were collected, including age, race, and ethnicity (non‐Hispanic White, non‐Hispanic Black/Hispanic/other), sex (male, female), education level (≤high school degree, some college, and college degree), marital status (married/partnered, single, and widowed/divorced/separated), annual household income (<$50,000, ≥$50,000), employment status (working, retired, and unemployed/disabled), and insurance status (private, Medicare, and other). Neighborhood deprivation status was calculated using the Area Deprivation Index, a measure comprised of 17 income, education, employment, and housing quality metrics determined at the census block level using 12‐digit Federal Information Processing Standard codes.^21^ Cancer‐related characteristics were also self‐reported, including cancer type, cancer stage (early, metastatic), years from diagnosis (<5, 5–10, and ≥10), age at diagnosis, current treatment status (active, nonactive), and travel time to the cancer provider (0–30, 31–60, and >60 min).

### 2.4 Statistical analysis

Descriptive survey statistics were calculated using medians and interquartile ranges (IQR) for continuous variables and frequencies and percentages for categorical variables. Bivariate analyses were conducted using Cramer's *V* and Cohen's *d* to estimate the in‐sample magnitude of associations.^22^ Exploratory modified Poisson models with robust standard errors^23^ estimating risk of trial‐related financial hardship were fitted to compute adjusted relative risks (aRR) and 95% confidence intervals (CI). The use of modified Poisson regression with robust errors for the analysis of binary outcomes produces reliable estimates of relative risk, which is more easily interpretable than the odds ratios produced by logistic regression.^23^ Due to sample size limitations,^24^ a subset of covariables for exploratory analysis in our regression models was selected a priori. Models included race, annual household income, employment status, insurance status, and travel time to cancer provider. Analyses were performed using SAS© software, version 9.4 (SAS Institute, Cary, NC).

## 3 RESULTS

Of the 650 survey respondents (21% response rate), 18% (*n* = 118) reported ever participating in a cancer clinical trial. Survey respondents were 47% Black or a person of color, 69% had annual household incomes <$50,000, 45% were unemployed or disabled, and 32% were Medicaid enrollees, dual eligible for Medicare and Medicaid, or uninsured. Compared to those who did not participate, respondents who participated in a clinical trial more often traveled >1 h to receive cancer care (31% vs. 15%; *V* = 0.21), had later‐stage cancer (64% vs. 49%; *V* = 0.13), and were more often college educated (47% vs. 35%; *V* = 0.1, Table S2). Respondents who reported participating in a cancer clinical trial were a median age of 57 (IQR 46–65), 52% Non‐Hispanic White, 47% with a college degree, 64% with annual household incomes <$50,000, 50% unemployed or disabled, and 46% Medicare beneficiaries (Table 1). Respondents were also most often diagnosed with breast cancer (59%), 47% were <5 years from diagnosis, 38% had metastatic cancer, 69% were on active treatment when surveyed, and 31% traveled more than an hour to receive cancer care. Notably, of patients who did not participate in a cancer clinical trial (*n* = 532), 13% declined participation due to cost.

**TABLE 1:** Respondent sociodemographics and clinical characteristics (*N* = 118).

	Overall (*N* = 118)	Did not report financial hardship (*n* = 63)	Reported financial hardship (*n* = 55)	*V*
	*n* (%)	*n* (%)	*n* (%)	
Age at survey, years (median, IQR)	57 (46–65)	55 (44–65)	58 (49–65)	*d* = 0.16
Race and ethnicity				
Non‐Hispanic White	61 (52)	33 (52)	28 (51)	0.01
Black/Hispanic/Other	57 (48)	30 (48)	27 (49)	
Sex				
Female	99 (84)	54 (86)	45 (82)	0.05
Male	19 (16)	9 (14)	10 (18)	
Education level				
≤High school degree	19 (16)	11 (17)	8 (15)	0.05
Some college	43 (36)	22 (35)	21 (38)	
≥College degree	56 (47)	30 (48)	26 (47)	
Marital status				
Married/partnered	51 (43)	28 (44)	23 (42)	0.05
Single	36 (31)	18 (29)	18 (33)	
Widowed/divorced/separated	29 (25)	16 (25)	13 (24)	
Missing	2 (2)	1 (2)	1 (2)	
Annual household income				
<$50,000	75 (64)	42 (67)	33 (60)	0.07
≥$50,000	43 (36)	21 (33)	22 (40)	
Employment status				
Working	36 (31)	22 (35)	14 (25)	0.15
Retired	23 (19)	14 (22)	9 (16)	
Unemployed/disabled	59 (50)	27 (43)	32 (58)	
Insurance status				
Private/Tricare	28 (24)	18 (29)	10 (18)	0.15
Medicare	54 (46)	25 (40)	29 (53)	
Dual eligible/Medicaid/other/none	36 (31)	20 (32)	16 (29)	
On active treatment				
No	37 (31)	22 (35)	15 (27)	0.08
Yes	81 (69)	41 (65)	40 (73)	
Cancer type				
Breast	70 (59)	37 (59)	33 (60)	0.12
Hematologic	37 (31)	22 (35)	15 (27)	
Other	11 (9)	4 (6)	7 (13)	
Years since cancer diagnosis				
<5 years	56 (47)	32 (51)	24 (44)	0.14
5–10 years	33 (28)	19 (30)	14 (25)	
>10 years	29 (25)	12 (19)	17 (31)	
Cancer stage				
Early stage	43 (36)	25 (40)	18 (33)	0.08
Metastatic	45 (38)	22 (35)	23 (42)	
Other	30 (25)	16 (25)	14 (25)	
Age at diagnosis				
18–39	31 (26)	18 (29)	13 (24)	0.15
40–59	62 (53)	29 (46)	33 (60)	
60 and older	25 (21)	16 (25)	9 (16)	
Travel time to cancer provider				
0–30 min	41 (35)	29 (46)	12 (22)	0.33
31–60 min	41 (35)	23 (37)	18 (33)	
>60 min	36 (31)	11 (17)	25 (45)	
Neighborhood deprivation				
Low	89 (75)	46 (73)	43 (78)	0.07
High	11 (9)	7 (11)	4 (7)	
Missing	18 (15)	10 (16)	8 (15)	

**FIGURE 1:**
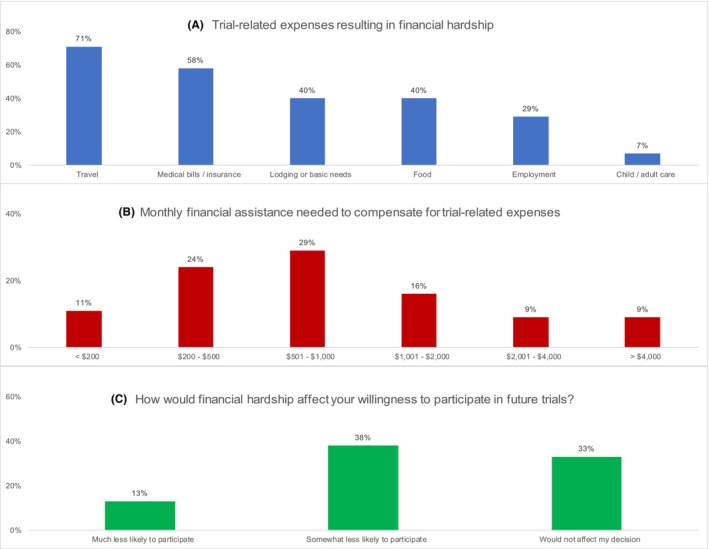
(A–C) Outcomes for respondents who reported trial‐related financial hardship (*N* = 55), including (A) Trial‐related expenses resulting in financial hardship, (B) Monthly financial assistance needed to compensate for trial‐related expenses, and (C) How financial hardship would affect willingness to participate in future trials.

Of respondents who reported participating in a cancer clinical trial, 47% (*n* = 55) reported financial hardship as a result of their trial participation. Respondents reporting trial‐related financial hardship were more often unemployed or disabled (58% vs. 43%; *V* = 0.15), Medicare enrolled (53% vs. 40%; *V* = 0.15), and traveled >1 h to their cancer provider (45% vs. 17%; *V* = 0.33) compared to respondents reporting no hardship (Table 1). Respondents who experienced trial‐related financial hardship most often reported expenses from travel (reported by 71% of respondents), medical bills or insurance (58%), dining out (40%), or housing needs (40%; Figure 1A).

Modeling results indicated that respondents traveling >1 h vs. ≤30 min to their cancer provider had a 2.2 times higher risk of financial hardship, even after adjusting for respondent race, income, employment, and insurance status (aRR = 2.24, 95% CI 1.31–3.83; Table 2). Respondents most often reported needing $501–$1000 per month to compensate for trial‐related expenses (29%), with 16% needing between $1001 and $2000 per month, and one in five (18%) needing more than $2000 monthly (Figure 1B). Over half (51%) of respondents reported less willingness to participate in future clinical trials due to incurred financial hardship (Figure 1C).

**TABLE 2:** Risk of financial hardship by patient sociodemographic and clinical characteristics (*N* = 118).

	Adjusted relative risk (95% confidence interval)
Race and ethnicity	
Non‐Hispanic White	(ref)
Black/Hispanic/Other	1.25 (0.86, 1.83)
Annual household income	
<$50,000	(ref)
≥$50,000	1.34 (0.93, 1.93)
Employment status	
Working	(ref)
Retired	0.98 (0.52, 1.86)
Unemployed/disabled	1.18 (0.73, 1.90)
Insurance status	
Private/tricare	(ref)
Medicare	1.51 (0.89, 2.55)
Dual eligible/Medicaid/other/none	1.17 (0.64, 2.14)
Travel time to cancer provider	
0–30 min	(ref)
31–60 min	1.44 (0.80, 2.60)
>60 min	2.24 (1.31, 3.83)

## 4 DISCUSSION

This cross‐sectional study of a diverse sample of patients receiving support from PAF found cancer clinical trial‐related financial hardship, most often stemming from travel expenses, affects almost half of trial‐enrolled patients. Though financial hardship is a well‐documented adverse effect of standard‐of‐care cancer treatment,^25^, ^26^, ^27^, ^28^, ^29^ this is one of the few studies to report on and quantify financial hardship and its impact on patients with lower incomes considering or participating in cancer clinical trials. Additionally, this study provides data on measures of both subjective financial hardship, or affordability, and objective financial hardship, or actual costs, as both are important in understanding potential financial barriers to clinical trial participation. For example, 40% of respondents in our sample with household incomes >$50,000 reported trial‐related subjective financial hardship, pointing to potential trial‐related financial barriers for patients who may not initially seem at risk.

Our study revealed high direct (medical bills and insurance) and indirect (travel and dining out) costs associated with cancer clinical trial participation, with one in five respondents reporting spending at least $2000 per month while on trial. Though substantial, costs of clinical trial participation are mostly unknown and rarely discussed with patients during the enrollment process, potentially due to difficulties surrounding the calculation of the actual amount patients will owe out of pocket for participation. Trial‐related costs are often expensed through multiple sources. The trial sponsor will usually cover the costs of the investigational agent and other direct “research” costs. If the patient is insured, “routine” care received while on trial is covered for nongrandfathered privately insured patients (via the Patient Protection and Affordable Care Act^13^), Medicaid enrollees (via the Clinical Treatment Act of 2020^30^), and Medicare beneficiaries. However, the language surrounding “routine” versus “research” costs for patients participating in trials is murky. Patients are often billed for items, such as blood work or scans while on trial treatment, which insurance providers deem as a research cost, even though the same blood work or scans are done as part of routine care. This lack of cost transparency is potentially why 58% of respondents in our sample reported medical bills or insurance costs as contributing to their experience of trial‐related financial hardship. Because cancer‐related financial hardship is associated with adverse physical and mental outcomes,^31^ an increased understanding of actual out‐of‐pocket trial costs and trial‐related cost transparency is needed during the trial enrollment process for the safety of patients participating in clinical trials.

In our sample, travel‐related expenses were the most frequently reported financial hardship stemming from cancer clinical trial participation, with almost three of four participants reporting financial burdens as a result of traveling to receive trial treatment. This result was confirmed in adjusted models, with patients traveling further to receive cancer care having more than twice the risk of financial hardship compared to those traveling shorter distances. Notably, we do not know if the cancer provider referred to in the survey is also where the respondent received their cancer clinical trial. However previous research has shown that compared to those with higher incomes, patients with cancer who have low incomes may be more likely to live in areas with less health care or clinical trial resources, and thus be required to travel farther distances to a site that offers cancer clinical trials. For example, Borno and colleagues found patients with lower incomes traveled a mean of 238 miles to participate in a clinical trial, compared to just 49 and 43 miles for those with middle and high incomes, respectively.^16^ Increased travel time to receive care may also result in more indirect expenses related to participating in a clinical trial. A study of patients with hematologic cancer participating in a clinical trial found 60% and 23% recommended financial reimbursement for travel and lodging costs, respectively.^32^ No matter the directionality, travel‐related expenses represent a larger barrier to clinical trial participation and retention that should be considered when designing financial interventions.

Our study revealed more than one in 10 respondents declined cancer clinical trial participation due to costs. These findings align with those by Unger and colleagues, who found patients with cancer who have low household incomes are less likely to participate in clinical trials.^10^, ^11^ As previously discussed, a lack of cost transparency exists surrounding clinical trial participation. Thus, at least some patients considering enrollment may not be presented with the full opportunity to understand the potential costs of participation, which may impact decision‐making. Furthermore, of respondents who participated in a cancer clinical trial, half reported less willingness to participate in future clinical trials due to incurred expenses. This may be due to the excessively high out‐of‐pocket costs associated with participation. For respondents in our study who participated in a cancer clinical trial, almost half reported needing over $1000 per month to compensate for trial‐related expenses. It is important to note that our respondents were those receiving financial services from PAF, and thus may be biased towards having lower incomes than those found in routine cancer care samples. However, our results are similar to those of Huey and colleagues, which found 48% of patients enrolled in a phase I oncologic trial had out‐of‐pocket costs of at least $1000 per month.^17^ Because current estimates suggest only 55% of eligible patients enroll on cancer clinical trials and 10%–15% of trial‐enrolled patients do not complete the trial,^33^, ^34^, ^35^ addressing financial hardship during routine cancer clinical trial enrollment and delivery could aid in increasing both recruitment to and retention in clinical trials.

As cost represented a major barrier to both current and future cancer clinical trial participation in our sample, interventions are needed to address baseline financial hardship for trial‐eligible patients and trial‐related financial hardship for trial‐enrolled patients. In contrast to commercially‐sponsored trials, which are increasingly providing stipends or financial reimbursements for their trial participants, publicly‐sponsored trials do not often include financial support. In 2018, a Congressional Request was directed to the National Cancer Institute to expand access to cancer clinical trials via “a new pilot initiative to investigate the impact of providing navigation and direct patient expense reimbursement associated with participation in cancer clinical trials on cancer clinical trial enrollment, retention, patient outcomes, and research outcomes, including among underrepresented and minority communities.”^36^ While an Ad Hoc Working Group on Clinical Trials Enrollment and Retention was formed and a report with detailed recommendations was generated,^37^ to our knowledge, no pilot intervention has originated from this request. However, two previous studies at academic medical centers have developed and tested interventions to address financial barriers to cancer clinical trial participation. Both studies implemented patient navigator‐supported reimbursements for trial‐related transportation and lodging expenses, which resulted in increased cancer clinical trial enrollment compared to preintervention trends.^38^, ^39^ Both studies were also run with support from foundation funding, which is an unsustainable funding mechanism for larger, nationwide changes to the clinical trial landscape. Due to the clear Federal interest surrounding diversifying cancer clinical trial participation, as well as the successes of previous interventions, we argue that the National Cancer Institute should prioritize funding more pilot projects testing financial interventions to increase and diversify clinical trial participation across multiple sites with diverse patient populations nationwide. This research may now be even more feasible based on a November 2023 announcement from the NIH Office of Extramural Research which detailed new “Allowable Costs Related to Participant [Clinical Trial] Inclusion Activities.”^40^ This announcement describes items for patient reimbursement that investigators are allowed to charge to their grant, such as money spent on travel to the research study site, meals during time spent on research activities, childcare, clinical research services, or financial incentives like gift cards. These data could strengthen the case for policy‐level interventions addressing financial barriers to clinical trial participation.

This study should be considered within the context of several limitations. Our collected survey data does not allow comparison of reported trial‐related financial hardship to potential financial hardship experienced by patients not participating in a clinical trial and instead receiving standard‐of‐care therapies. Furthermore, results from this study may not generalize to the broader population of patients with cancer or patients with more resources, since respondents were cancer survivors who previously received help accessing or paying for care from PAF. Thus, our sample is specifically enriched to provide data on individuals most at risk for trial‐related financial hardship and results should be interpreted accordingly. Because 53% of our sample were more than 5 years from their cancer diagnosis, recall bias may exist regarding financial hardship experienced while on a clinical trial. Questions regarding cancer clinical trials did not specify the type of trial, such as therapeutic trials or behavioral trials. Our sample may be biased towards individuals able to navigate services from a nonprofit organization or access web‐based surveys.

## 5 CONCLUSION

In a sample of individuals with cancer who have experienced cost‐related barriers to cancer care, clinical trial‐related financial hardship affected almost half of trial‐enrolled patients. Financial hardship most often stemmed from travel expenses, and over half of respondents reported less willingness to participated in future trials due to costs. To decrease inequitable access to cancer clinical trials, as well as increase the pace of scientific discovery, interventions are needed to reduce adverse financial participation effects and potentially improve cancer clinical trial participation for all patients.

## AUTHOR CONTRIBUTIONS

**Courtney P. Williams:** Conceptualization (equal); data curation (equal); formal analysis (equal); methodology (equal); writing – original draft (equal); writing – review and editing (equal). **Luqin Deng:** Formal analysis (equal); writing – review and editing (equal). **Nicole E. Caston:** Data curation (equal); writing – review and editing (equal). **Kathleen Gallagher:** Data curation (equal); project administration (equal); writing – review and editing (equal). **Rebekah Angove:** Data curation (equal); project administration (equal); writing – review and editing (equal). **Maria Pisu:** Supervision (equal); writing – review and editing (equal). **Andres Azuero:** Methodology (equal); writing – review and editing (equal). **Rebecca Arend:** Conceptualization (equal); writing – review and editing (equal). **Gabrielle B. Rocque:** Conceptualization (equal); supervision (equal); writing – review and editing (equal).

## CONFLICT OF INTEREST STATEMENT

None.

## PRIOR PRESENTATIONS

This study was presented as a poster at the 2023 ASCO Quality Care Symposium, October 27–28, 2023, Boston, MA.

## PRECIS

In this survey of individuals with cancer who previously received financial assistance from a nonprofit organization, cancer clinical trial‐related financial hardship affected 47% of patients who reported participating in a clinical trial. Respondents who experienced trial‐related financial hardship most often reported expenses from travel, and over half of respondents reported less willingness to participate in future clinical trials due to incurred financial hardship.

## Supporting information


Appendix S1.

## Data Availability

The data that supports the findings of this study are available on request from the corresponding author. The data are not publicly available due to privacy or ethical restrictions.
