# Supplementary material for: Understanding the financial cost of cancer clinical trial participation
Source: Cancer Med. 2024 Apr 17;13(8):e7185. doi: 10.1002/cam4.7185 (PMC11022148; doi:10.1002/cam4.7185)
Supplement: Supplementary file 1 — Appendix S1. [file CAM4-13-e7185-s001.docx]

Supplementary Figure S1. Patient Advocate Foundation survey tool.

Clinical Trials Micro Survey - CW UAB

Survey Flow

Block: Introduction & Consent Language (2 Questions)

Standard: Eligibility General (3 Questions)

Standard: Eligibility Cancer Diagnosis (5 Questions)

Standard: Clinical Trial Financial Q (6 Questions)

Standard: Clinical Trial Attitudes (1 Question)

Standard: Demographics - ADI to be added (10 Questions)

Standard: Qualitative Interview Q (1 Question)

Start of Block: Introduction & Consent Language

Q1.1 *You are being asked to complete an online research survey from Patient Advocate Foundation. We are conducting this research because we want to learn more about the financial challenges experienced by patients when participating in clinical trials.
This survey was deemed exempt by the University of Alabama at Birmingham Institutional Review Board. Completing this survey is optional and your answers will be kept anonymous. By beginning this survey, you are providing consent to participate in this research.
 This survey will take* ***less than 10 minutes*** *of your time. Please mark only one answer for each question unless otherwise indicated.*

**Q1.2 I agree to participate in this survey.**

- Yes (1)
- No (0)

Skip To: End of Survey If Q1.2 = No

End of Block: Introduction & Consent Language

Start of Block: Eligibility General

**Q2.1 Are you at least 18 years of age?**

- Yes (1)
- No (0)

Skip To: End of Survey If Q2.1 = No

**Q2.2 How old are you today?** 
(enter a number of years) __________

**Q2.3 Where do you live?**

- United States (1)
- Another country besides United States (2)

Skip To: End of Survey If Q2.3 = Another country besides United States

End of Block: Eligibility General

Start of Block: Eligibility Cancer Diagnosis

**Q3.1 Have you ever been diagnosed with cancer?**

- Yes (1)
- No (0)

Skip To: End of Survey If Q3.1 = No

**Q3.2 Please select the primary cancer you were diagnosed with from the list below.**

- Bladder (1)
- Bone (2)
- Breast (3)
- Cervical (4)
- Colon or Rectal (5)
- Endocrine (6)
- Gastrointestinal (7)
- Genitourinary (8)
- Gynecologic (9)
- Head and Neck (10)
- Hepatocellular carcinoma (11)
- Hodgkin Lymphoma (12)
- Leukemia (13)
- Liver (14)
- Lung (15)
- Melanoma (16)
- Multiple Myeloma (17)
- Neurological (18)
- Non-Hodgkin Lymphoma (19)
- Ocular (20)
- Ovarian (21)
- Prostate (22)
- Sarcoma (23)
- Skin (24)
- Thyroid (25)
- Other (26) __________________________________________________

**Q3.3 How many years ago were you first diagnosed with this cancer?**

- Less than 1 year ago (1)
- 1-5 years ago (2)
- 5-10 years ago (3)
- More than 10 years ago (4)

**Q3.4 Which of the following statements most accurately describes you?**

- I have been diagnosed with early stage cancer (1)
- I have been diagnosed with metastatic cancer (cancer that has spread to other parts of the body) (2)
- Other (3)

**Q3.5 Within the past six weeks, have you had any of the following?**
 *Please select all that apply.*

- Surgery for cancer (1)
- Chemotherapy or any other injectable medication for cancer (2)
- Medications taken by mouth to treat cancer or to prevent cancer from returning (3)
- ⊗None of the above (4)

End of Block: Eligibility Cancer Diagnosis

Start of Block: Clinical Trial Financial Q

**Q4.1 Have you ever participated in a cancer clinical trial?**

- Yes (1)
- No (0)

Skip To: Q4.6 If Q4.1 = No

**Q4.2 Has participating in a clinical trial resulted in financial hardship (e.g., high out-of-pocket costs, worry about finances) for you and your family?**

- Not at all (1)
- A little bit (2)
- Somewhat (3)
- Quite a bit (4)
- Very much (5)

Skip To: End of Block If Q4.2 = Not at all

**Q4.3 What trial-related expenses were a financial hardship for you and your family?**
*Please select all that apply.*

- ⊗None (1)
- Travel (e.g., gas, parking) (2)
- Food (e.g., eating out away from home) (3)
- Child/adult care (e.g., hiring sitters or paying for daycare) (4)
- Employment (e.g., unpaid time off work, lost wages) (5)
- Medical bills/insurance (e.g., copays, coinsurance, deductibles) (6)
- Lodging or basic needs (groceries, utilities, rent/mortgage) (7)

Skip To: End of Block If Q4.3 = None

**Q4.4 What amount of financial assistance would have compensated for your clinical trial-related medical and non-medical expenses?**

- <$200/month (1)
- $200-$500/month (2)
- $501-$1,000/month (3)
- $1,001-$2,000/month (4)
- $2001-$4,000/month (5)
- >$4,000/month (6)

**Q4.5 How would financial hardship affect your willingness to participate in future trials?**

- Much less likely to participate (1)
- Somewhat less likely to participate (2)
- Would not affect my decision whether or not to participate (3)
- Somewhat more likely to participate (4)
- Much more likely to participate (5)

Display This Question:

If Q4.1 = No

**Q4.6 Did you decline clinical trial enrollment due to the associated costs of participating (e.g., transportation to clinic visits, extra medical bills, having to take time off work)?**

- Yes (1)
- No (0)

End of Block: Clinical Trial Financial Q

Start of Block: Clinical Trial Attitudes

**Q5.1 For the next questions, please mark the number indicating how much you agree or disagree with each of the statements about clinical trials: (1 = Strongly Disagree 7 = Strongly Agree)**

| 1 = Strongly Disagree 7 = Strongly Agree | 1 | 2 | 3 | 4 | 5 | 6 | 7 |
| --- | --- | --- | --- | --- | --- | --- | --- |
| I’d get improved cancer treatment if I took part in a clinical trial (1) |  |  |  |  |  |  |  |
| People who join clinical trials have a better chance of beating their cancer (2) |  |  |  |  |  |  |  |
| Joining a clinical trial would mean I’d receive the best existing cancer treatment (3) |  |  |  |  |  |  |  |
| By joining a clinical trial, I would receive better health care (4) |  |  |  |  |  |  |  |
| Taking part in a clinical trial is a lot more trouble than just getting the usual treatment (5) |  |  |  |  |  |  |  |
| Getting treatment in a clinical trial is less safe than getting the usual cancer treatment (6) |  |  |  |  |  |  |  |
| Treatments received in a clinical trial could be unsafe for myself (7) |  |  |  |  |  |  |  |
| My taking part in a clinical trial could lead to more health problems (8) |  |  |  |  |  |  |  |
| Joining a clinical trial would make cancer treatment more difficult (9) |  |  |  |  |  |  |  |
| In general, people should know more about clinical trials (10) |  |  |  |  |  |  |  |
| Clinical trials are of little importance to me (11) |  |  |  |  |  |  |  |
| Access to cancer treatment clinical trials is important to me (12) |  |  |  |  |  |  |  |
| People who take part in clinical trials are helping all of us fight cancer (13) |  |  |  |  |  |  |  |
| I feel certain my safety would be watched closely in a clinical trial (14) |  |  |  |  |  |  |  |
| Doctors and nurses tell patients the truth about what to expect during a clinical trial (15) |  |  |  |  |  |  |  |
| If I took part in a clinical, I would be treated like a guinea pig (16) |  |  |  |  |  |  |  |
| Doctors and nurses mislead their patients who are involved in clinical trials (17) |  |  |  |  |  |  |  |
| It would be safe for me to join a clinical trial for treatment (18) |  |  |  |  |  |  |  |
| I will get my needed treatment as soon as possible if I am in a clinical trial (19) |  |  |  |  |  |  |  |
| If I am in a clinical trial I will have to spend extra time having more tests and doctor visits (20) |  |  |  |  |  |  |  |
| I may have to spend more time and money on transportation and childcare and may lose income due to time away from work if I participate in a clinical trial (21) |  |  |  |  |  |  |  |

End of Block: Clinical Trial Attitudes

Start of Block: Demographics - ADI to be added

Q6.1 FINALLY, PLEASE TELL US MORE ABOUT YOURSELF:

**Please indicate your gender identity:**

- Cisgender Female (assigned female at birth and identify as female) (1)
- Cisgender Male (assigned male at birth and identify as male) (2)
- Transgender Female (assigned male at birth and identify as female) (3)
- Transgender Male (assigned female at birth and identify as male) (4)
- Genderqueer or Non-Binary (neither exclusively male nor female) (5)
- Another gender category (6)
- I choose not to disclose (7)
- Non-binary / third gender (8)
- Prefer not to say (9)

**Q6.2 What is your race?**
*Check all that apply.*

- American Indian/Alaska Native (1)
- Asian (2)
- Black/African American (3)
- Native Hawaiian or Other Pacific Islander (4)
- White/Caucasian (5)
- Other (6) __________________________________________________

**Q6.3 Are you of Hispanic or Latino(a) origin or descent?**

- Yes (1)
- No (0)

**Q6.4 Please indicate your highest level of academic achievement:**

- Less than a high school diploma or equivalent (1)
- High school diploma or equivalent (2)
- Some college, no degree (3)
- Associate’s degree (4)
- Bachelor’s degree (5)
- Master’s or doctoral degree (6)

**Q6.5 Please choose the option that best describes your current marital status.**

- Single (1)
- Married/Living with partner (2)
- Partnered/Not living together (3)
- Widowed (4)
- Divorced (5)
- Separated (6)
- Other (7) __________________________________________________

**Q6.6 Please indicate your current household income per year in US dollars.**

- Less than $20,000 (1)
- $20,000 to $34,999 (2)
- $35,000 to $49,999 (3)
- $50,000 to $74,999 (4)
- $75,000 to $99,999 (5)
- More than $100,000 (6)

**Q6.7 What is your current employment status?**
Please select what you consider to be your main activity.

- Working full time (≥32 hours/week) (1)
- Working part time (1-31 hours/week) (2)
- Unemployed, looking for work (3)
- Unemployed, not looking for work (4)
- In job training (5)
- Temporarily laid off (no pay) (6)
- Retired (7)
- On short term disability (8)
- On long term disability (9)
- Permanently disabled (10)
- I do not work (11)
- In school (12)
- Other (13)

**Q6.8 Please indicate your health insurance type.**
 *Please select all that apply.*

- Private (e.g. BlueCross, Cigna) (1)
- Medicare (2)
- Medicaid (3)
- Tri-Care/Other Military (4)
- Indian Health Service (5)
- The Veterans Health Administration (VA) (6)
- ⊗None (7)
- Other (8)

**Q6.9 How old were you when you were first diagnosed with cancer?**

- Under 18 (1)
- 18-29 (2)
- 30-39 (3)
- 40-49 (4)
- 50-59 (5)
- 60-69 (6)
- 70 or older (7)

**Q6.10 How long does it take for you to travel to an appointment to see your cancer provider?**

 If you have more than one cancer provider, please indicate the time it takes for you to travel to an appointment with the **provider you see the most frequently.**

- Less than 15 minutes (1)
- 15-30 minutes (2)
- 30-60 minutes (3)
- 60-90 minutes (4)
- More than 90 minutes (5)

End of Block: Demographics - ADI to be added

Start of Block: Qualitative Interview Q

Display This Question:

If Q4.2 = Not at all

**Q7.1 Would you be interested in being contacted by the research team at UAB about participating in a 30-60 minute conversation about clinical trial financial challenges? You would be compensated for this activity.**

- Yes (1)
- No (2)

End of Block: Qualitative Interview Q

Supplementary Table S2. Respondent sociodemographics and clinical characteristics (N=650).

|  | **Overall**  **(N=650)** | **Did not participate in a clinical trial**  **(n=532)** | **Participated in a clinical trial**  **(n=118)** | **V** |
| --- | --- | --- | --- | --- |
|  | **n (%)** | **n (%)** | **n (%)** |  |
| Age at survey, years (median, IQR) | 57 (48-64) | 57 (49-64) | 57 (46-65) | d=0.09 |
| Race and ethnicity |  |  |  | 0.01 |
| Non-Hispanic White | 346 (53) | 285 (54) | 61 (52) |  |
| Black / Hispanic / Other | 304 (47) | 247 (46) | 57 (48) |  |
| Sex |  |  |  | 0.01 |
| Female | 548 (84) | 449 (84) | 99 (84) |  |
| Male | 102 (16) | 83 (16) | 19 (16) |  |
| Education level |  |  |  | 0.10 |
| ≤ High school degree | 137 (21) | 118 (22) | 19 (16) |  |
| Some college | 272 (42) | 229 (43) | 43 (36) |  |
| ≥ College degree | 241 (37) | 185 (35) | 56 (47) |  |
| Marital status |  |  |  | 0.04 |
| Married / Partnered | 269 (41) | 218 (41) | 51 (43) |  |
| Single | 185 (28) | 149 (28) | 36 (31) |  |
| Widowed / Divorced / Separated | 187 (29) | 158 (30) | 29 (25) |  |
| Missing | 9 (1) | 7 (1) | 2 (2) |  |
| Annual household income |  |  |  | 0.06 |
| < $50,000 | 449 (69) | 374 (70) | 75 (64) |  |
| ≥ $50,000 | 201 (31) | 158 (30) | 43 (36) |  |
| Employment status |  |  |  | 0.05 |
| Working | 218 (34) | 182 (34) | 36 (31) |  |
| Retired | 142 (22) | 119 (22) | 23 (19) |  |
| Unemployed / Disabled | 290 (45) | 231 (43) | 59 (50) |  |
| Insurance status |  |  |  | 0.08 |
| Private / Tricare | 190 (29) | 162 (30) | 28 (24) |  |
| Medicare | 249 (38) | 195 (37) | 54 (46) |  |
| Dual eligible / Medicaid / Other / None | 210 (32) | 174 (33) | 36 (31) |  |
| Missing | 1 (0) | 1 (0) | 0 (0) |  |
| On active treatment |  |  |  | 0.04 |
| No | 182 (28) | 145 (27) | 37 (31) |  |
| Yes | 468 (72) | 387 (73) | 81 (69) |  |
| Cancer type |  |  |  | 0.17 |
| Breast | 461 (71) | 391 (73) | 70 (59) |  |
| Hematologic | 114 (18) | 77 (14) | 37 (31) |  |
| Other | 75 (12) | 64 (12) | 11 (9) |  |
| Years since cancer diagnosis |  |  |  | 0.14 |
| < 5 years | 402 (62) | 346 (65) | 56 (47) |  |
| 5-10 years | 132 (20) | 99 (19) | 33 (28) |  |
| > 10 years | 116 (18) | 87 (16) | 29 (25) |  |
| Cancer stage |  |  |  | 0.13 |
| Early stage | 316 (49) | 273 (51) | 43 (36) |  |
| Metastatic | 220 (34) | 175 (33) | 45 (38) |  |
| Other | 114 (18) | 84 (16) | 30 (25) |  |
| Age at diagnosis |  |  |  | 0.07 |
| 18-39 | 130 (20) | 99 (19) | 31 (26) |  |
| 40-59 | 376 (58) | 314 (59) | 62 (53) |  |
| 60 and older | 144 (22) | 119 (22) | 25 (21) |  |
| Travel time to cancer provider |  |  |  | 0.21 |
| 0-30 minutes | 360 (55) | 319 (60) | 41 (35) |  |
| 31-60 minutes | 176 (27) | 135 (25) | 41 (35) |  |
| > 60 minutes | 114 (18) | 78 (15) | 36 (31) |  |
| Neighborhood deprivation |  |  |  | 0.04 |
| Low | 450 (69) | 361 (68) | 89 (75) |  |
| High | 73 (11) | 62 (12) | 11 (9) |  |
| Missing | 127 (20) | 109 (20) | 18 (15) |  |
